# Supplementary material for: Synthesis and Characterization of Block Copolymers of Poly(silylene diethynylbenzen) and Poly(silylene dipropargyl aryl ether)
Source: Polymers (Basel). 2021 May 7;13(9):1511. doi: 10.3390/polym13091511 (PMC8125851; doi:10.3390/polym13091511)
Supplement: Supplementary file 1 [file polymers-13-01511-s001.zip › polymers-1195940-supplementary.pdf]

Supporting Information

Synthesis and characterization of block copolymers of poly(silylene arylacetylene) and poly(silylene dipropargyl aryl ether)

Man Gao [1], Chengyuan Shang [2], Jixian Li [1], Gang Han [1], Junkun Tang [1], Qiaolong Yuan [1] and Farong Huang [1,*]

[1] Key Laboratory of Specially Functional Polymeric Materials and Related Technology (Ministry of Education), School of Materials Science and Engineering, East China University of Science and Technology, Shanghai 200237, China; mangao96@163.com (M.G.); chmxy521@163.com (J.L.); ganghmail@163.com (G.H.); jktang@ecust.edu.cn (J.K.); qlyuan@ecust.edu.cn (Q.Y.)

[2] Research and Application Center for Structural Composites, Aerospace Research Institute of Materials & Processing Technology, Beijing 100076, China; shangchengyuan@163.com

* Correspondence: fhuanglab@ecust.edu.cn

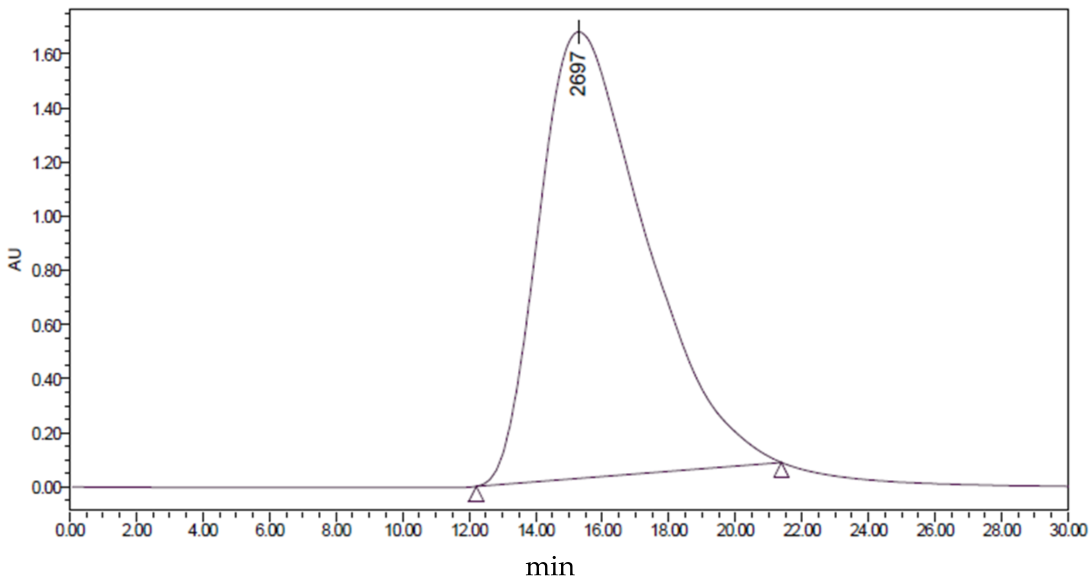

Figure S1. Gel permeation chromatography of AAA.

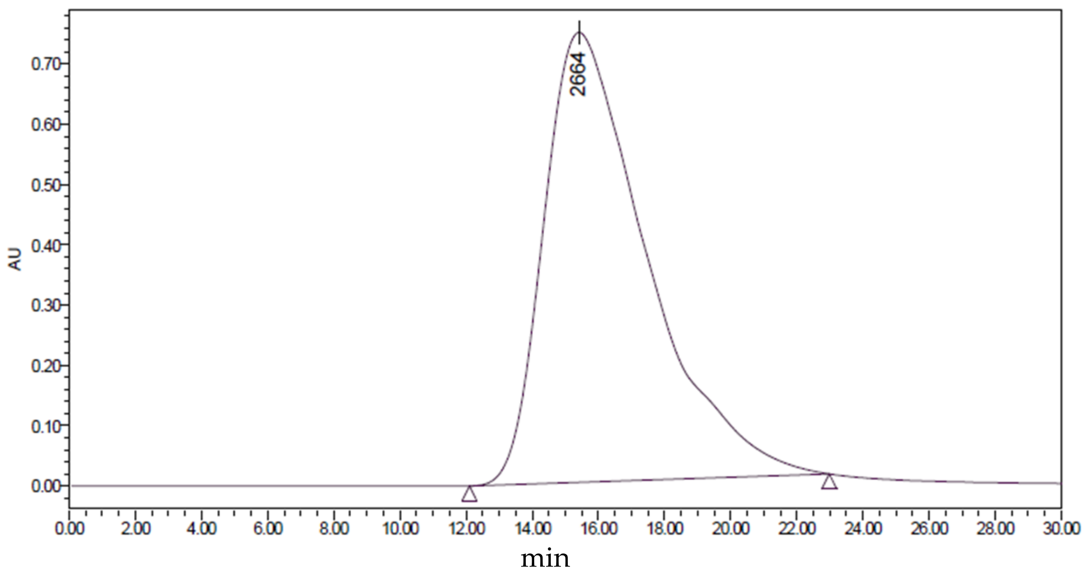

Figure S2. Gel permeation chromatography of ABA-A.

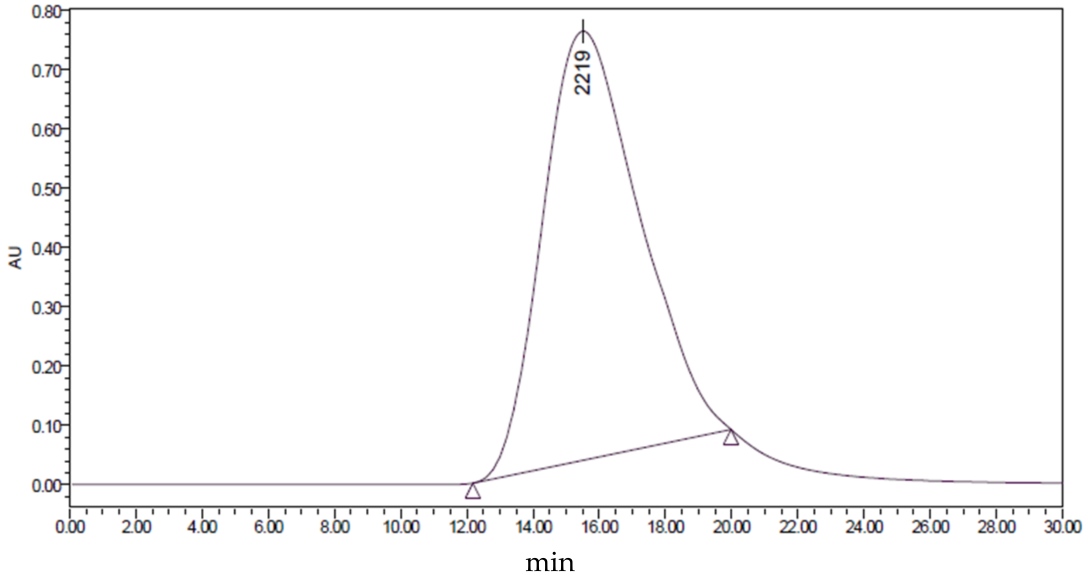

Figure S3. Gel permeation chromatography of ABA-O.

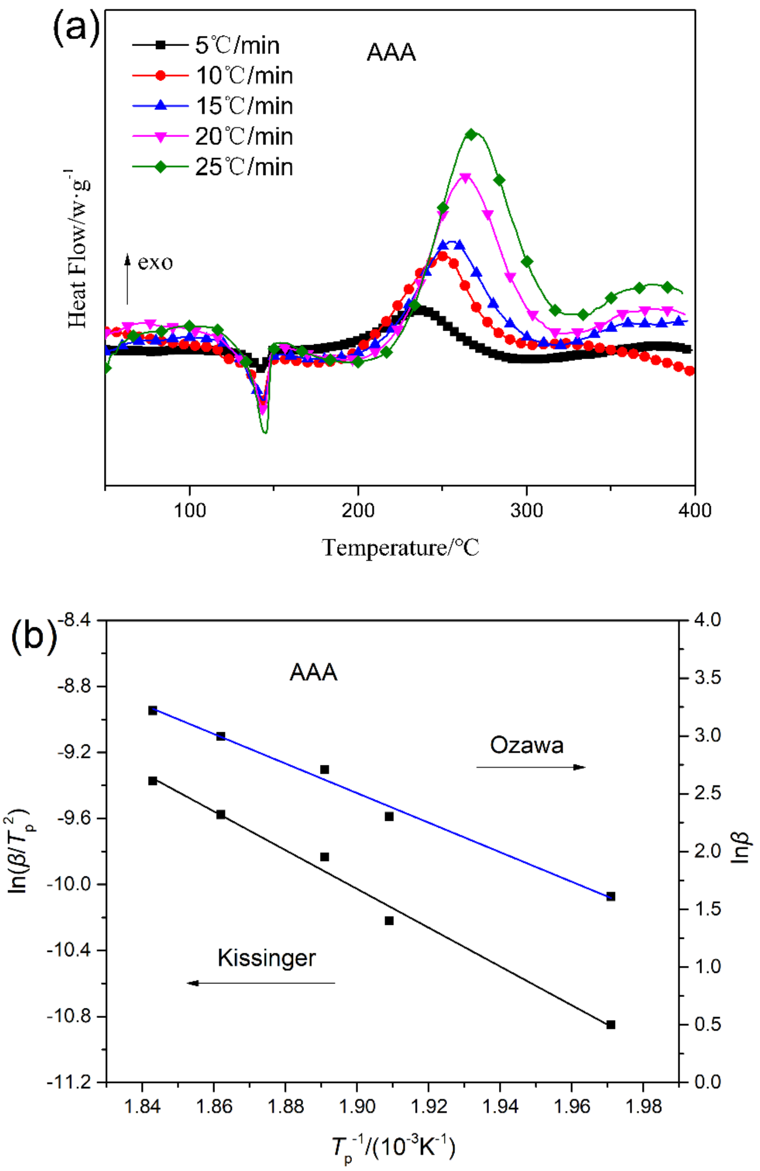

Figure S4. (a) DSC curves of the AAA polymer at different heating rates; (b) the relationship of $\ln(\beta/T_P^2)$ versus $1/T_P$ and $\ln(\beta)$ versus $1/T_P$.

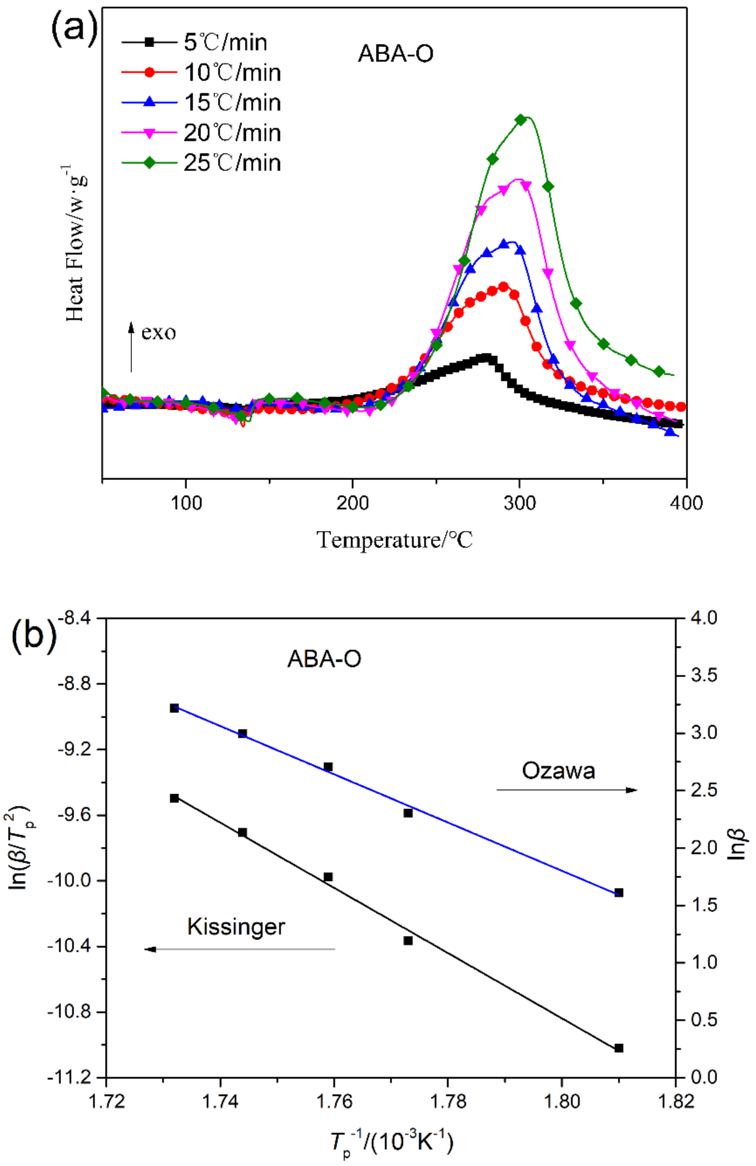

Figure S5. (a) DSC curves of the ABA-O copolymer at different heating rates; (b) the relationship of $\ln(\beta/T_P^2)$ versus $1/T_P$ and $\ln(\beta)$ versus $1/T_P$.
